# Towards a Precision Medicine Approach Based on Machine Learning for Tailoring Medical Treatment in Alkaptonuria

**DOI:** 10.3390/ijms22031187

**Published:** 2021-01-26

**Authors:** Ottavia Spiga, Vittoria Cicaloni, Anna Visibelli, Alessandro Davoli, Maria Ausilia Paparo, Maurizio Orlandini, Barbara Vecchi, Annalisa Santucci

**Affiliations:** 1 Department of Biotechnology, Chemistry and Pharmacy, University of Siena, 53100 Siena, Italy; anna.visibelli@student.unisi.it (A.V.); maurizio.orlandini@unisi.it (M.O.); annalisa.santucci@unisi.it (A.S.); 2 Toscana Life Sciences Foundation, 53100 Siena, Italy; v.cicaloni@toscanalifesciences.org; 3 Hopenly s.r.l., 41058 Vignola, Italy; alessandrodavoli@hopenly.com (A.D.); ausiliapaparo@hopenly.com (M.A.P.); barbara@hopenly.com (B.V.)

**Keywords:** alkaptonuria, rare disease, machine learning, precision medicine, data analysis, QoL scores

## Abstract

ApreciseKUre is a multi-purpose digital platform facilitating data collection, integration and analysis for patients affected by Alkaptonuria (AKU), an ultra-rare autosomal recessive genetic disease. It includes genetic, biochemical, histopathological, clinical, therapeutic resources and quality of life scores that can be shared among registered researchers and clinicians in order to create a Precision Medicine Ecosystem (PME). The combination of machine learning application to analyse and re-interpret data available in the ApreciseKUre shows the potential direct benefits to achieve patient stratification and the consequent tailoring of care and treatments to a specific subgroup of patients. In this study, we have developed a tool able to investigate the most suitable treatment for AKU patients in accordance with their Quality of Life scores, which indicates changes in health status before/after the assumption of a specific class of drugs. This fact highlights the necessity of development of patient databases for rare diseases, like ApreciseKUre. We believe this is not limited to the study of AKU, but it represents a proof of principle study that could be applied to other rare diseases, allowing data management, analysis, and interpretation.

## 1. Introduction

Precision medicine (PM) is an emerging approach for disease prevention, diagnosis and treatment that takes into account individual variability in genes, environment, proteomics, metabolomics and lifestyle [1]. The capacity to collect, harmonize and analyse data streams is the core for developing a “Precision Medicine Ecosystem” (PME) in which biochemical and clinical resources are shared between researchers, clinicians and patients [2] and can constitute useful guides to generate an exhaustive and dynamic picture of the individual, to identify new potential biomarkers and to tailor a medical treatment suitable for every patient. In PM context, multimedia data management plays a key role not only for common pathologies, but especially for rare disorders, where patients are scattered around the world.

In particular, Alkaptonuria (AKU) is an ultra-rare autosomal recessive metabolic disease [3] with a very low prevalence (1:1,000,000–250,000) [4], caused by mutation in the structure of homogentisate 1,2-dioxygenase (HGD) [4], an enzyme involved in the metabolism of tyrosine and phenylalanine. The deficient activity of HGD enzyme leads to the accumulation of Homogentisic Acid (HGA), which undergoes oxidation and polymerization, forming a dark-brown pigmentation in different connective tissues with a phenomenon called “ochronosis”. Such pigmentation involves mostly the osteoarticular tissues leading to a serious arthropathy with tissues degeneration, chronic inflammation and oxidative stress [5]. The deposition of the dark pigment involves skin, salivary glands [5], brain [6] and cardiac system [7,8], but the most damaged tissues are bone and cartilage [9]. Moreover, recent studies have classified AKU as a secondary amyloidosis [7,8], characterised by deposition of serum amyloid A (SAA) fibers, which is a circulating protein produced at high levels (100–1000 times the normal plasmatic condition of about 4–6 mg/L) in chronic inflammation, making SAA a sensitive biomarker of inflammation. Another marker linked to chronic inflammation is chitotriosidase (CHIT1), a chitinase mainly expressed in the differentiated and polarized macrophages. Therefore, in AKU, besides inflammation, patients also suffer from significant oxidative stress caused by high systemic levels of HGA and its products. In this context, Protein Thiolation index (PTI) interestingly denotes and summarizes the oxidative state of AKU patients. One of the main problems in carrying out clinical research on AKU is the lack of a standardized methodology to assess disease severity and response to treatment, which is complicated by the large variety of AKU symptoms from an individual to another. A reliable way to monitor patients’ clinical condition and overall health status is the use in clinical practice and research of measures of Quality of Life (QoL) scores.

To overcome the limitations due to the scarcity of specimens and data available for AKU and the wide range of AKU symptoms, we have recently established a comprehensive digital ecosystem, ApreciseKUre, that integrates patient-derived information (QoL scores, lifestyle), clinician-derived information (urine, blood, plasma analysis), mutational analysis (genotypes, protein stability) and therapeutic treatments offering an exhaustive visualization of different informative layers, to support clinicians and researchers in a PM approach to AKU [10,11,12,13,14,15]. The ApreciseKUre database can be a good starting point for the creation of a new clinical management tool in AKU, which will lead to the development of a deeper knowledge network on the disease and will advance its treatment [10,11,12].

The integration of quality of life scores with clinical and therapeutic data will have a central role in order to create a complete PME, supporting clinicians to tailor a medical treatment to every AKU patient. AKU can be treated symptomatically during the early stages (generally using anti-inflammatories, painkillers, low protein diet and vitamin C) whereas, for end stages, total joint and heart valve replacements may be required. Currently, there is no specific therapy for AKU, although a clinical trial with nitisinone is in progress. Moreover, it has been already proved that both methotrexate and anti-oxidants have an excellent efficacy to inhibit the production of amyloid in AKU model chondrocytes [16,17]. Our integrated platform, jointly with a machine learning analysis, described in this study, will be useful to achieve an AKU patients stratification and in monitoring the evolution of biomarkers and QoL scores to tailor the most suitable treatment to each patients sub-group.

**Figure 1:**
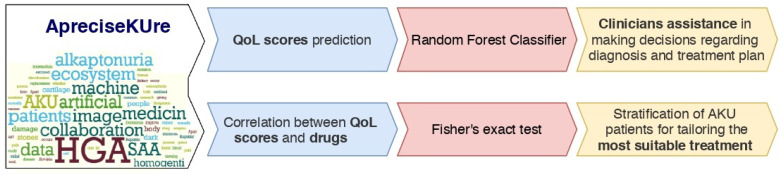
Workflow scheme represented by two stages, ’Quality of Life (QoL) scores prediction’ in the top and ’Correlation between QoL scores and drugs’ in the bottom.

The workflow of our study is summarized in Figure 1. The first goal of this work was the prediction of the QoL scores based on both personal and clinical AKU patients’ information collected in ApreciseKUre. A fine-tuned scoring system can indeed assist clinicians in making sound decisions regarding diagnosis and treatment plan. Then, it was better investigated the correlation between the values of the QoL scores and the drugs the patients take. This could pave the way to stratify AKU patients and to tailor the most suitable treatment to each patient sub-group in a typical PM perspective. Tailoring treatment to the patient has become a promising approach for maximizing efficacy and minimizing drug toxicity and it is not trivial in an ultra-rare disease like AKU. We believe that this AKU-dedicated preliminary study can represent a proof of principle applicable not only to other rare diseases, but it could be also valuable to larger research communities with an increasing number of affected patients.

## 2. Materials and Methods

### 2.1. Dataset

The ApreciseKUre (http://www.bio.unisi.it/aku-db/) contains data from 203 patients, of whom 129 do not contain missing data (for a full description of ApreciseKUre see Supplementary Materials S1). Each patient in the ApreciseKUre database is characterized by more than 100 features (for the complete list see Supplementary Materials S1), describing biochemical (i.e., SAA, CHIT1 and PTI), clinical, genotypic information and replies to questionnaires evaluating QoL scores. It has been performed patients assessment involving 11 QoL scores: (i) physical health score (PHS), (ii) mental health score (MHS); (iii) AKU Severity Score Index (AKUSSI) for joint pain (AJP) and (iv) AKUSSI spinal pain (ASP); (v) Knee injury and Osteoarthritis Outcome Score (KOOS) pain (KOOSp), (vi) KOOS symptoms (KOOSs), (vii) KOOS daily living (KOOSdl), (viii) KOOS sport (KOOSsp), (ix) KOOS QOL; (x) Health Assessment Questionnaire Disability Index (HAQ-DI) and (xi) global pain visual analog scale (hapVAS) (for more details about each score, see supplementary materials in [14]). Moreover, it includes information about drugs taken. We decide to divide the drugs in painkillers, anti-inflammatories and others; then, we group them in several sub-categories:

1. painkillers: opioid, paracetamol, metamizole;
2. anti-inflammatories: Non-steroidal anti-inflammatory drugs (FANS), corticosteroid;
3. others: antiacid, antiarhythmic, antiasthma, antibiotic, anticoagulant, anticonvulsant, antidepressant, antiglaucoma, antigout, antihistamine, antihyperglycemic, antihypertensive, antimuscarinic, antiosteoporotic, antiparkinson, antipsychotic, antireumatic, antiviral, calcium, cholesterol-lowering medication, corticosteroid, diuretic, hormone, methotrexate, proton pump inhibitor, skeletal muscle relaxant, sodium chloride, thyroid hormones, vitamins.

The amount of data used in the analysis varies according to the information available for each QoL score: in particular, we have 134 to 138 rows of data at our disposal, depending on the particular QoL score we are focusing on.

### 2.2. Machine Learning Classification

The first goal of this work has been the prediction of the QoL scores based on different patients information collected in ApreciseKUre. Because of the small amount of available data, we decided to turn these scores into categorical variables; for each of them, in particular, we divided its range in three equally spaced regions denoted by 0, 1 and 2, corresponding to decreasing severity of health conditions. Given a specific QoL score, we defined *y* as the vector representing its values (one for each patient): the *k*-th element $y_{k}$ could then take value 0, 1 or 2. The prediction was performed with a one-vs-all approach: one of three classes was chosen (let *i* represent its value), and the new vector $y^{\left(i\right)}$ was defined such that its *k*-th element is:

(1) $$y_{k}^{\left(i\right)}\equiv \left\{\begin{array}{c} 1\;\mathrm{if}\;\;y_{k}=i\\ 0\;\mathrm{if}\;\;y_{k}\neq i \end{array}\right.\;.$$

The prediction for $y^{\left(i\right)}$ turned out to be a standard binary classification, which was carried out using the Random Forest (RF) algorithm [18,19], an ensemble classifier that uses multiple decision trees to obtain a better prediction performance. It creates many classification trees and a bootstrap sample technique is used to train each tree from the set of training data.Finally, to evaluate the performance of the model, we defined the usual elements of the confusion matrix, i.e., true positive (TP), true negative (TN), false positive (FP) and false negative (FN) as:

(2a) $$\begin{array}{cc} \; & {\mathrm{TP}}^{\left(i\right)}\equiv \sum_{k}\delta_{{\hat{y}}_{k}^{\left(i\right)},y_{k}^{\left(i\right)}}\;\delta_{y_{k}^{\left(i\right)},1} \end{array}$$

(2b) $$\begin{array}{cc} \; & {\mathrm{TN}}^{\left(i\right)}\equiv \sum_{k}\delta_{{\hat{y}}_{k}^{\left(i\right)},y_{k}^{\left(i\right)}}\;\delta_{y_{k}^{\left(i\right)},0} \end{array}$$

(2c) $$\begin{array}{cc} \; & {\mathrm{FN}}^{\left(i\right)}\equiv \sum_{k}\left[1-\delta_{{\hat{y}}_{k}^{\left(i\right)},y_{k}^{\left(i\right)}}\right]\;\delta_{y_{k}^{\left(i\right)},1} \end{array}$$

(2d) $$\begin{array}{cc} \; & {\mathrm{FP}}^{\left(i\right)}\equiv \sum_{k}\left[1-\delta_{{\hat{y}}_{k}^{\left(i\right)},y_{k}^{\left(i\right)}}\right]\;\delta_{y_{k}^{\left(i\right)},0}\;, \end{array}$$

where δ is the Kronecker delta and ${\hat{y}}_{k}^{\left(i\right)}$ is the prediction for $y_{k}^{\left(i\right)}$. Once the elements of the confusion matrix were computed, we introduced other standard metrics such as:

1. accuracy:
  (3) $${\mathrm{acc}}^{\left(i\right)}\equiv \frac{{\mathrm{TP}}^{\left(i\right)}+{\mathrm{TN}}^{\left(i\right)}}{{\mathrm{TP}}^{\left(i\right)}+{\mathrm{TN}}^{\left(i\right)}+{\mathrm{FP}}^{\left(i\right)}+{\mathrm{FN}}^{\left(i\right)}}\;;$$
2. recall:
  (4) $${\mathrm{recall}}^{\left(i\right)}\equiv \frac{{\mathrm{TP}}^{\left(i\right)}}{{\mathrm{TP}}^{\left(i\right)}+{\mathrm{FN}}^{\left(i\right)}}\;;$$
3. precision:
  (5) $${\mathrm{prec}}^{\left(i\right)}\equiv \frac{{\mathrm{TP}}^{\left(i\right)}}{{\mathrm{TP}}^{\left(i\right)}+{\mathrm{FP}}^{\left(i\right)}}\;;$$
4. $F_{1}$ score:
  (6) $$F_{1}^{\left(i\right)}\equiv \frac{{2\mathrm{TP}}^{\left(i\right)}}{2{\mathrm{TP}}^{\left(i\right)}+{\mathrm{FP}}^{\left(i\right)}+{\mathrm{FN}}^{\left(i\right)}}\;;$$
5. Matthews correlation coefficient (MCC) [20]:
  (7) $${\mathrm{MCC}}^{\left(i\right)}\equiv \frac{{\mathrm{TP}}^{\left(i\right)}\times {\mathrm{TN}}^{\left(i\right)}-{\mathrm{FP}}^{\left(i\right)}\times {\mathrm{FN}}^{\left(i\right)}}{\sqrt{\left({\mathrm{TP}}^{\left(i\right)}+{\mathrm{FP}}^{\left(i\right)}\right)\left({\mathrm{TP}}^{\left(i\right)}+{\mathrm{FN}}^{\left(i\right)}\right)\left({\mathrm{TN}}^{\left(i\right)}+{\mathrm{FP}}^{\left(i\right)}\right)\left({\mathrm{TN}}^{\left(i\right)}+{\mathrm{FN}}^{\left(i\right)}\right)}}\;.$$

Among these, the most appropriate metric to be considered was the MCC, as it is the least sensitive to the case of imbalanced classes [21,22]; by definition, it varies over the range (−1,1), with the value 0 corresponding to random guess.

Given that the index *i* takes three values, i=0,1,2, we ended up with three values for each of these metrics, corresponding to the number of 2-combinations of three elements. In order to derive a single value, different ways of computing a mean value were possible (e.g., macro-, micro- and weighted-average); in particular, we used a weighted average, and defined:

(8) $$m\equiv \sum_{i=1}^{3}m^{\left(i\right)}\;w_{i}\;,\;w_{i}\equiv \frac{{\mathrm{TP}}^{\left(i\right)}+{\mathrm{FN}}^{\left(i\right)}}{\dim y}\;,$$

where *m* generically stands for one of the metrics in Equations (3)–(7); each class, then, was weighted by the number of positive instances with respect to the total.

### 2.3. Techniques in Determining Correlation

The second goal of the present work has been to look for a correlation between the values of the QoL scores and the drugs the patients take. Clearly, it is not uncommon for these 33 drugs to be taken in different combinations: for this reason, we treated each of them as a binary variable (with value equal to 1 if it is taken by the patient, to 0 otherwise), and each record has been characterized by a 33-dimensional vector representing if the patient takes a particular drug or not. The problem of looking for a possible correlation between the drugs and the QoL scores then became that of studying the correlation between two categorical variables. Therefore various methodologies were accessed and compared (see Supplementary Section S2 for a detailed discussion).

## 3. Results

### 3.1. Quality of Life Scores Prediction

The first goal of this work has been the prediction of the QoL scores based on different patients information collected in ApreciseKUre, both personal (e.g., date of birth, gender, country of origin, etc.) and clinical (e.g., inflammation biomarkers, results from blood tests, etc.).

For this purpose, we have considered all the QoL scores with the exception of PHS and MHS. Each score takes real values, with KOOS being the only one where large values correspond to good health conditions (absence of pain).

In order to carry out the classification, we used the RF algorithm [18,19]; by comparing its performance against that of logistic regression (LR) and support vector machine (SVM) [23], it turned out the be the one giving the best results.

The hyperparameters of the RF were optimized with the Python library Hyperopt in order to maximize (the absolute value of) the MCC, with a training-validation spitting of 0.8–0.2. We optimized the following hyperparameters: max depth ($d_{\max}$), max features ($f_{\max}$), min samples leaf ($sl_{\min}$), min samples split ($ss_{\min}$), number of estimators ($N_{\mathrm{estim}}.$); we report in Table 1 the results for the different QoL scores.

Given the limited amount of data, we adopted the following procedure for training and testing: once the hyperparameters had been optimized, we performed *M*=50 independent trainings and tests, each time with a different training-test splitting, with the training size randomly chosen between 0.7 and 0.8; we then computed an average on all the metrics obtained in each iteration. The results of the prediction are given in Table 2, together with the number of records available for each QoL score; in particular, for each metric, both the mean value (μ) and the standard deviation (σ) are shown.

As can be seen, the prediction algorithm performs best for KOOS daily living, KOOS sport, KOOS symptoms and KOOS QOL: despite the rather limited amount of data, about 70% of the records where correctly classified for these QoL scores. If, in the future, information from new patients is recorded, we expect these results to improve significantly.

### 3.2. Correlation between Drugs and Quality of Life Scores

The second goal of the present work has been to look for a correlation between the values of the QoL scores and the drugs the patients take, grouped in the sub-categories, as explained before. We decided to perform Fisher’s exact test on all the combinations QoL score vs. drug, using the software R, employing the Benjamini-Hochberg procedure to deal with multiple comparisons. Out of the 33 drugs we considered in the analysis, it turns out that 8 of them showed significant correlation with at least one QoL score; we report a summary of the results in Table 3, where we simply indicate whether a given drug is correlated with a given QoL score. It is important to notice that “no” does not mean that the drug and the QoL score are uncorrelated, but simply that there is not a significant evidence of correlation; in the future, with a larger amount of data available, it is possible that those drug will turn out to be correlated.

**Figure 2:**
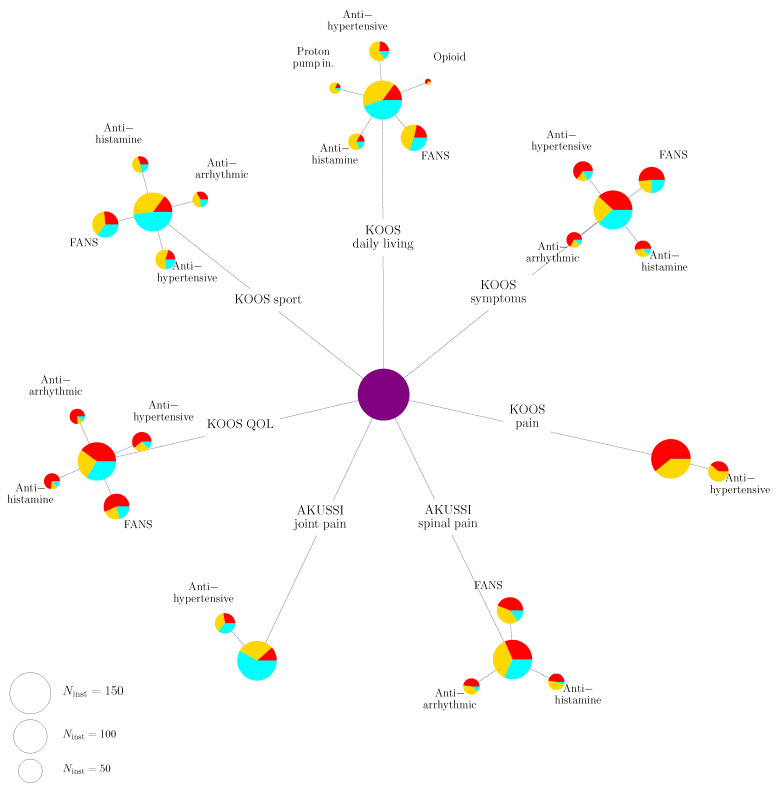
Dense results of the Fisher’s exact test. For each QoL score, a first level of pie charts is shown, representing the psycho-physical state of the patients (from red to cyan, corresponding to progressively better health conditions); the area of each circle is proportional to the number of patients for which the information about that QoL score is available. A second level of pie charts, then, shows the impact of drugs on that particular QoL score, with the same conventions as before. As a reference, we also show three benchmark circles whose sizes correspond to the case where the number of patients is 150, 100 and 50, respectively.

The full results of the Fisher’s exact test can be found in Supplementary Table S3, together with the threshold (shown in the last column) used to accept or reject the null hypothesis, computed according to the Benjamini-Hochberg procedure with a false discovery rate *Q* set to Q=0.2; the drugs which show a significant correlation are highlighted with bold characters. Moreover, a dense representation is shown in Figure 2.

In Figure 2, for each QoL score a first pie chart is represented, whose dimension is proportional to the number of patients for which there is information for that given QoL score. The colours are divided according to the psycho-physical state of the patient: from red (bad health conditions) to cyan (absence of pain). In the second level of pie charts, only the drugs for which evidence of correlation has been found are shown. The area of the circle is proportional to the number of patients taking that drug for that given QoL score. As a reference, we also show the size of the circles corresponding to three benchmark values for the number of patients, i.e., 150, 100 and 50.

## 4. Discussion

The first goal of this work is the prediction of QoL scores in AKU patients. Our previous studies showed that, in a rare and multisystemic disease like AKU, QoL scores help to identify health needs and to evaluate the impact of disease, suggesting the presence of a correlation between QoL and the clinical data deposited in the ApreciseKUre database, which could be instrumental in shading light on AKU complexity. Here, we have developed machine learning applications that perform a prediction of the QoL scores based on data deposited in the ApreciseKUre. In particular, it is based on information about the patients, both personal (date of birth, gender, country of origin, etc.), biochemical and clinical (e.g., amyloidosis, oxidative stress and inflammation biomarkers, results from blood and urine tests, etc.). In this analysis, we consider 9 QoL scores: AKUSSI joint pain, AKUSSI spinal pain, KOOS pain, KOOS symptoms, KOOS daily living, KOOS sport, KOOS QOL, HAQ-DI and hapVAS. Because of the small amount of available data, we decide to turn these scores into three categorical variables (0, 1 and 2) corresponding to decreasing severity of health conditions (i.e., 0 is the worst condition and 2 is the best condition). The classification was carried out using the RF algorithm and comparing its performance against LR and SVM in order to obtain the best result which were then validated. In accordance with our previous study, [14], the algorithm prediction performs best for KOOS daily living, KOOS sport and KOOS symptoms. In fact, despite the rather limited amount of data, about 70% of the records where correctly classified. Thus, our model suggested that KOOS indicator could be a useful tool to better understand symptoms and difficulties experienced by AKU patients. Indeed, KOOS is a valid, reliable and responsive instrument to evaluate both short-term and long-term consequences of knee injury and primary osteoarthritis (OA). It is a patient-reported outcome measurement, developed to assess the opinion of patients about their knees and associated problems, and it is routinely used for follow-up evaluations [24]. KOOS prediction could be important to assess consequences of primary OA, to evaluate changes from week to week induced by treatment (such as medication, surgery, physical therapy etc.) or over the years due to a primary knee injury, post-traumatic OA or primary OA [24], to identify the main important prognostic biomarkers of AKU, to help the clarification of physio-pathological mechanisms of AKU and ochronosis, and to assess the efficacy of future pharmacological treatments. The second goal of this study is the investigation of the correlation between QoL scores and drugs taken by AKU patients. Similarly to the majority of rare genetic diseases, the existing state-of-the-art treatment for AKU is unsatisfactory. To date AKU has no licensed therapy and treatment is symptomatic. Generally, for end-stages joint and heart valve, replacement surgery is required. Previously suggested approaches included a low protein diet for reducing the amount of tyrosine and phenylalanine intake and hence HGA production. Thanks to this attitude, lower values of HGA in blood and urines have been detected especially for children [25].However, in AKU the low-tyrosine dietary strategy was found not always effective, only palliative and also difficult to follow without the supervision of a specialist and it cannot be performed for prolonged times. The idea of adapting diet or treatment according to “personal” factors (such as age, gender, physiological state, or physical activity and QoL scores) and to pathological features (need to follow a low level-protein diet), as well as to special conditions (such as risk of disease) is common today. We believe that our tool could be effective to investigate the most suitable therapy in accordance with QoL scores, which indicates changes in quality of life of patients before/after a specific treatment. Being AKU related to chronic inflammation, oxidative stress and amyloidosis, symptomatic treatments are based on anti-inflammatories (FANS, corticosteroid, FANS+corticosteroid), anti-oxidant (such as Vitamin C) and painkillers (opioid, paracetamol and metamizole). AKU is also linked to cardiovascular ochronosis [26]. Ochronosis is associated with aortic valve stenosis but mitral and pulmonary valves can be affected as well. Numerous case reports have suggested that cardiovascular calcification and stenosis may be associated with pigment deposition in the aortic and mitral valves, endocardium, pericardium, aortic intima, and coronary arteries. In this context, antiarrhythmic and antihypertensive agents could help AKU patients to improve AKU conditions, as obtained by the application of our method. As well as FANS and opioid resulted to be particularly effective in reducing AKU pain as suggested by a high correlation with KOOS scores, HAQ-DI, hap-VAS. Also, common drugs not related to specific AKU symptoms, such as cholesterol lowering and proton pomp inhibitors, showed a correlation with some QoL scores. In the case of vitamins, they resulted to be effective in the only case of KOOS pain evaluation.

## 5. Conclusions

In conclusion, our study could be summarized in two main goals

1. Prediction of the QoL scores based on both personal and clinical AKU patients’ information collected in ApreciseKUre.
2. The investigation of the correlation between the values of the QoL scores and the drugs the patients take.

The previously described bioinformatics approach could pave the way to achieve AKU patient stratification and to tailor the most suitable treatment to each patient sub-group in a typical PM perspective. This AKU-dedicated preliminary study can represent a proof of principle useful not only to other rare diseases, but it could be also valuable to more common diseases with a larger cohort of patients.

## Figures and Tables

**Table 1:** List of optimized hyperparameters for RF used in the analysis, for the different QoL scores.

QoL Score	$d_{\max}$	$f_{\max}$	${\mathrm{sl}}_{\min}$	${\mathrm{ss}}_{\min}$	$N_{\mathrm{estim}}$
AKU joint pain	10	0.718	6	8	53
AKU spinal pain	23	0.990	27	14	72
KOOS pain	1	0.718	5	17	51
KOOS symptoms	10	0.609	21	17	94
KOOS daily living	2	0.554	25	44	78
KOOS sport	23	0.663	23	52	56
KOOS QOL	6	0.554	10	25	80
hapVAS	24	0.554	24	13	38

**Table 2:** QoL scores prediction with RF; the last column represents the number of records available for that particular QoL score.

	Accuracy		Precision		Recall		$F_{1}$		MCC		*N*
	μ	σ	μ	σ	μ	σ	μ	σ	μ	σ	
AKU joint pain	0.669	0.052	0.530	0.082	0.593	0.061	0.530	0.071	0.180	0.095	138
AKU spinal pain	0.589	0.046	0.327	0.111	0.440	0.065	0.342	0.084	0.037	0.101	138
KOOS pain	0.648	0.064	0.487	0.084	0.547	0.077	0.495	0.085	0.204	0.127	134
KOOS symptoms	0.657	0.070	0.543	0.111	0.585	0.089	0.542	0.102	0.235	0.147	134
KOOS daily living	0.718	0.044	0.553	0.064	0.623	0.061	0.578	0.061	0.346	0.089	134
KOOS sport	0.689	0.049	0.415	0.086	0.546	0.073	0.464	0.079	0.275	0.096	130
KOOS QOL	0.662	0.050	0.463	0.129	0.509	0.076	0.460	0.090	0.232	0.112	134
hapVAS	0.571	0.054	0.371	0.136	0.359	0.086	0.325	0.098	0.066	0.127	136
HAQ-DI	0.624	0.084	0.624	0.104	0.624	0.084	0.596	0.096	0.163	0.183	138

**Table 3:** Evidence of correlation between the drugs considered in this analysis and the QoL scores; while “yes" means that the correlation is significant, “no" indicates that with the available data there is no evidence of correlation.

	FANS	Antiarry-	Antihi-	Antihyper-	Cholesterol-	Opioid	Proton	Vitamins
		Thmic	Stamine	Tensive	Lowering		Pump in.	
AKUSSI	no	no	no	yes	no	no	no	no
joint pain								
AKUSSI	no	no	no	yes	no	no	no	no
spinal pain								
KOOS	yes	yes	yes	yes	no	yes	yes	yes
pain								
KOOS	no	no	no	yes	no	no	no	no
symptoms								
KOOS	yes	no	no	yes	yes	no	yes	no
daily living								
KOOS	yes	no	no	yes	no	yes	yes	no
sport								
KOOS	yes	no	no	yes	no	yes	yes	no
QOL								
HAQ-	yes	no	no	no	no	yes	yes	no
DI								
hap-	yes	no	no	no	no	yes	yes	no
VAS								

## Data Availability

ApreciseKUre Database available at: http://www.bio.unisi.it/aku-db/.

## Supplementary Materials

The following are available online at https://www.mdpi.com/1422-0067/22/3/1187/s1.
