# Supplementary material for: Towards a Precision Medicine Approach Based on Machine Learning for Tailoring Medical Treatment in Alkaptonuria
Source: Int J Mol Sci. 2021 Jan 26;22(3):1187. doi: 10.3390/ijms22031187 (PMC7865235; doi:10.3390/ijms22031187)
Supplement: Supplementary file 1 [file ijms-22-01187-s001.pdf]

Supplementary Materials:

S1. ApreciseKUre Database

ApreciseKUre (for registration see http://www.bio.unisi.it/) is a digital platform developed by using MySQL and php language and implemented for the collection/analysis of data deriving from AKU patients. The goal of this database is summarized in fig. S1:

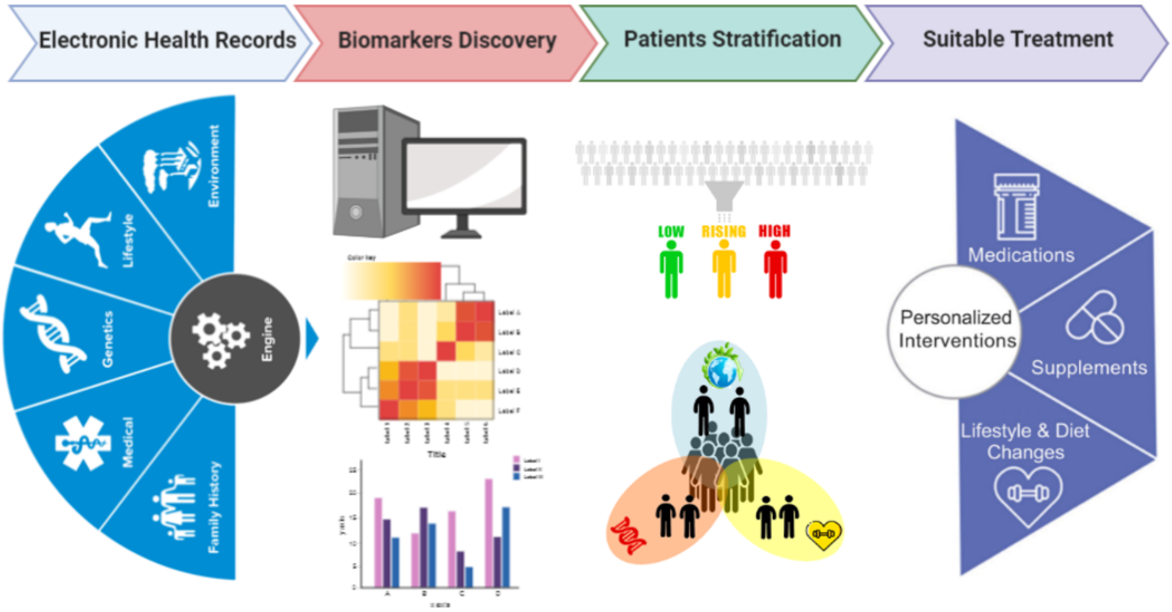

Supplementary Figure S1. ApreciseKUre: An integrated bioinformatics digital ecosystem platform for a rare disease to address –omics challenges in Precision Medicine

ApreciseKUre is an interactive online tool that includes an automatic system of data analysis. In the online platform it is in fact possible to compute correlations of the patients features (based on the Pearson correlation coefficient and p-value) and other statistical analysis. The available data are divided in several information layers which are: Demographics and Genetics, Oxidative stress, inflammation and amyloidosis biomarkers, Lifestyle, Quality of Life Scores, Concomitant Diseases, Drugs, Blood Analysis, Urine Analysis, Plasma Analysis, Histopathology. The database is composed of a large cohort of AKU patients for a total amount of 203 individuals:

- 25 patients from Associazione Italiana Malati diAlkaptonuria;
- 39 patients derived from Suitability of Nitisinone In AKU (SONIA) 1, forming one of the 3 steps of the clinical trial called DevelopAKUre;
- 139 derived from SONIA 2, the second step of DevelopAKUre.

Every patient in the ApreciseKUre database is characterized by more than 100 features, describing clinical, genotypic and metabolic information. The study of this large cohort of patients could have important applications toward a Precision Medicine approach in order to tailor the most suitable treatment for each patient. The informational layers and the features are listed in the following list:

1. Demographics and Genetics
    - id_patient
    - id_in_hgd_gene_mutation_database
    - Gender
    - date_of_birth

- protein_change_allele_1
- dna_change_allele_1
- exon/intron_allele_1
- protein_change_allele_2
- dna_change_allele_2
- exon/intron_allele_2
- secondary_structure_allele_1
- specific_activity_allele_1
- specific_activity_allele_2
- Country
- Relatives
- father's_origin
- mother's_origin
- who_is_affected_in_family
- year_diagnosis_AKU

2. Oxidative stress, inflammation and amyloidosis biomarkers

- saa: Serum Amyloid A [microg/mL] (ELISA)
- chitotriosidase: chitotriosidase [nmol/h/ml] (ELISA)
- serum_hga: Homogentisic acid [microM] (ELISA)
- hga_hplc: Homogentisic acid [mmol/L] (HPLC)
- catd: Cathepsin D [ng/mL] (ELISA)
- il-6: Interleukin-6 [pg/mL] (ELISA)
- il-1beta: Interleukin-1 Beta [pg/mL] (ELISA)
- il-1ra: Interleukin Receptor antagonist [pg/mL] (ELISA)
- tnf_a: Tumor necrosis factor alfa [pg/mL] (ELISA)
- crp: C-reactive protein [mg/dL] (ELISA)
- crp_elisa: C-reactive protein [microg/mL] (ELISA)
- mmp3: Matrix metalloproteinase 3 [ng/mL] (ELISA)
- aopp: Advanced oxidation protein products [micromol/dL] (ELISA)
- PSH: Serum protein thiols [micromol]
- CySSP: Protein/cysteine mixed disulphides [micromol]
- CyGlySSP: Protein/cysteineglycine mixed disulphides [micromol]
- HcySSP: Protein/Homocysteine mixed disulphides [micromol]
- yGluCySSP: Protein/Gamma-Glutamylcysteine mixed disulphides [micromol]
- GSSP: Protein/Glutathione mixed disulphides [micromol]
- RSSP: S-thiolated proteins [micromol]
- PTI: Protein Thiolation Index

3. Lifestyle

- smoker/cigarettes_a_day: N/day
- alcohol_units_weekly: N/week
- BMI: Body Mass Index

4. Quality of Life Scores

- physical_health_score: Physical Health Score
- mental_health_score: Mental Health Score
- AKUSSI_jointpain: AKU Severity Score Index joint pain
- AKUSSI_spinalpain: AKU Severity Score Index spinal pain
- KOOSpain: Knee injury and Osteoarthritis Outcome Score pain
- KOOSsymptoms: Knee injury and Osteoarthritis Outcome Score symptoms
- KOOSdaily_living: Knee injury and Osteoarthritis Outcome Score daily living
- KOOSsport: Knee injury and Osteoarthritis Outcome Score sport
- KOOS_QOL: Knee injury and Osteoarthritis Outcome Score Quality of Life
- hapVAS: Global pain visual analog scale
- HAQ-DI: Health Assessment Questionnaire Disability Index

5. Concomitant Diseases

- arterial_hypertension: Y/N
- hyperlipoproteinemia: Y/N
- hypothyroidism: Y/N
- asthma: Y/N
- diabetes_mellitu: Y/N

- hypercholesterolemia: Y/N
- ochronotic_arthropathy: Y/N
- osteoporosis: Y/N
- other_diseases: Y/N

6. Drugs

- anti_inflammatories: Y/N
- ATC_antinflammatories: Anatomical Therapeutic Chemical Classification Code
- painkillers: Y/N
- ATC_painkillers: Anatomical Therapeutic Chemical Classification Code other_drugs: Y/N
- ATC_code: Anatomical Therapeutic Chemical Classification Code

7. Blood analysis

- glucose [mg/dL]
- creatinine [mg/dL]
- cholesterol [mg/dL]
- triglycerides [mg/dL]
- hdl_cholesterol [mg/dL]
- ldl_cholesterol [mg/dL]
- alkaline_phosphatase [UI/L]
- cystatin_C [mg/dL]

8. Urine Analysis

- urines_code [Code]
- date_urines [Date]
- creatinine_urines [mg/dl]
- hga_urines Homogentisic acid [mg/24h]
- bqa Benzoquinone acetic acid [mg/dl]
- urate Urate [mg/dl]
- uric_acid_urine Urine Acid [mg/dl]
- tyr_urines Tyrosine [mg/dl]
- hypoxanthine_urines Hypoxanthine urines [mg/dl]
- xanthine_urines Xanthine urines [mg/dl]

9. Plasma Analysis

- id_plasma [Code]
- date_plasma [Date]
- creatinine_plasma Creatinine in plasma [micromol/L]
- hga_plasma: Homogentisic acid [micromol/L]
- tyr: Tyrosine [micromol/L]
- phe: Phenylalanine [micromol/L]
- trp: Tryptophan[micromol/L]
- hypoxanthine: Hypoxanthine [micromol/L]
- xanthine: Xanthine [micromol/L]
- uridine: Uridine [micromol/L]
- uric_acid [micromol/L]

10. Histopathology

- congo_red: slide image
- alizarin_red: slide image
- oarsi_grade: slide image

S2. Statistical Techniques

The problem of looking for a possible correlation between the drugs and the QoL scores could be reduced to the study of the correlation between two categorical variables. The standard approach is to resort to a contingency table, where the number of times two particular values of the categorical variables occur are shown. For example, the contingency table between the KOOS pain and the corticosteroids is shown in table S2;

Supplementary Table S2. Example of contingency table: specifically, the one between KOOS pain and corticosteroids is shown.

Corticosteroids \ KOOS pain	0	1	2
0	18	39	56
1	2	11	8

the interpretation is straightforward: out of the patient which have a level of KOOS pain equal to 0, 18 of them do not take corticosteroids, whereas 2 do. The standard approach is then to compute the χ^2 between the two variables, under the null hypothesis for them to be uncorrelated; to this purpose, a matrix $N^{(\mathrm{obs})}$ of observations was computed; for the case of KOOS pain vs. corticosteroids, its element was given precisely by table S2, i.e.:

$$N^{(\mathrm{obs})} = \begin{pmatrix} 18 & 39 & 56 \\ 2 & 11 & 8 \end{pmatrix} . \tag{1}$$

Under the hypothesis of no correlation between the two variables, if we denote by n_{ij} the element of $N^{(\mathrm{obs})}$ corresponding to row i and column j, and by $n \equiv \sum_{i,j} n_{ij}$ the total number of observations, we defined a matrix $N^{(\mathrm{expec})}$ of expected occurrences, with elements $\tilde{n}_{ij}$ as:

$$\tilde{n}_{ij} = n\, p_{ij} \equiv \frac{1}{n} \sum_{k,l} n_{ik}\, n_{lj} \, . \tag{2}$$

For the example under exam, we got:

$$N^{(\mathrm{expec})} = \begin{pmatrix} 12.6 & 42.2 & 58.2 \\ 2.4 & 7.8 & 10.8 \end{pmatrix} . \tag{3}$$

Clearly, the rows and columns sum to the same values as in $N^{(\mathrm{expec})}$. We then proceeded with the computation of the χ^2, defined as:

$$\chi^2 = \sum_{i,j} \frac{\left(n_{ij} - \tilde{n}_{ij}\right)^2}{\tilde{n}_{ij}} , \tag{4}$$

and compared it to the tabulated values, or used it compute other quantities (e.g., the Cramér's V) to decide whether to accept or reject the null hypothesis. The problem with such an approach, however, is that, because of the limited amount of data, the matrix of expected occurrences has some elements with small values; in particular, if any of its elements are less than 5, the χ^2 approach is not appropriate (see, e.g., [1]), and others should be chosen. One alternative is the so-called Fisher's exact test [2], which directly computes the p-value; we then decided to perform such a test on all the combinations QoL score vs. drug, using the software R. For instance, the result for the previous example would be $p = 0.484$, resulting in the impossibility to reject the null hypothesis.

Another problem we had to deal with was that of multiple comparisons; since we perform many tests (for a given QoL score, one for each drug), it may happen that the null hypothesis is rejected just by chance: for example, if we choose 0.05 as threshold and if the null hypothesis is true for all the tests we perform, we expect 5% of them to have a p-value less or equal than 0.05, leading to a rejection of the null hypothesis in the 5% of cases just by chance. In order to cure this issue, different solutions have been proposed, such as the Bonferroni correction or the Benjamini-Hochberg procedure [3], and they usually lead to a redefinition of the threshold used to accept or reject the null hypothesis. We chose the

employ the Benjamini-Hochberg procedure: for each QoL score, all the tests were carried out, they were then sorted by the corresponding p-value, and the quantity

$$\frac{i}{m}\,Q \tag{5}$$

was computed, where i is the rank index, m is the number of tests performed and Q is called false discovery rate which has to be chosen. The p-value p_i corresponding to the largest i for which $p_i < i\,Q/m$ was chosen as a threshold for all the tests: a given test j leads to the rejection of the null hypothesis if $p_j < p_i$. We set $Q = 0.2$, as it is a standard choice in the case one wants to avoid many false negatives, as they could lead to important discoveries [1].

S3. Supplementary Tables

	FANS	anti-acid	antiarrhythmic	anti-asthma	anti-biotic	antico-agulant	anticon-vulsant	antide-pressant	anti-glaucoma	anti-gout	antihi-stamine	antihyper-glycemic
AKUSSI joint pain	0.129	0.212	0.499	1.000	0.347	1.000	1.000	0.197	0.420	0.013	0.488	1.000
AKUSSI spinal pain	0.043	0.449	0.309	0.210	0.293	0.853	0.119	1.000	1.000	0.309	0.215	0.210
KOOS pain	**0.008**	0.220	**0.015**	0.112	0.784	0.294	0.116	0.479	0.485	0.701	**0.042**	1.000
KOOS symptoms	0.072	0.189	0.060	0.470	1.000	0.054	0.228	1.000	0.470	0.300	0.307	1.000
KOOS daily living	**0.003**	1.000	0.022	0.127	0.141	0.153	0.435	0.403	0.552	0.112	0.173	1.000
KOOS sport	**0.001**	1.000	0.116	1.000	0.564	0.851	0.751	0.763	1.000	0.482	0.485	0.600
KOOS QOL	**0.006**	1.000	0.119	1.000	0.749	0.250	0.432	0.240	1.000	0.119	0.276	1.000
HAQ-DI	**0.040**	0.730	0.002	0.522	0.662	0.160	0.172	0.344	0.522	0.509	0.694	0.522
hap-VAS	**0.000**	0.525	0.525	0.640	0.776	1.000	0.332	0.340	0.309	1.000	0.734	0.640

	antihyper-tensive	antimus-carinic	antiosteo-porotic	antipsy-chotic	antireu-matic	anti-viral	calcium	cholesterol-lowering	cortico-steroid	diuretic	hormone
AKUSSI join pain	**0.001**	0.116	1.000	1.000	1.000	1.000	0.098	0.046	0.179	0.761	0.818
AKUSSI spina pain	**0.009**	0.529	0.698	1.000	0.529	0.529	1.000	0.279	0.432	0.548	0.043
KOOS pain	**0.017**	0.485	0.212	0.112	1.000	1.000	0.103	0.054	0.484	0.572	0.110
KOOS symptoms	**0.008**	0.470	1.000	0.470	1.000	1.000	0.284	0.452	0.079	0.293	0.153
KOOS daily living	**0.000**	0.127	0.238	0.127	1.000	1.000	0.114	**0.020**	0.176	0.186	0.069
KOOS sport	**0.003**	1.000	0.727	1.000	0.262	0.600	0.312	0.154	0.587	0.369	0.005
KOOS QOL	**0.001**	1.000	0.708	1.000	0.239	1.000	0.642	0.100	0.655	0.283	0.026
HAQ-DI	0.000	0.130	0.245	0.130	1.000	1.000	0.260	0.143	0.213	0.044	0.090
hap-VAS	0.007	0.640	1.000	0.640	0.309	0.309	0.615	0.110	0.172	0.548	0.463

	metamizole	metho-trexate	opioid	parace-tamol	proton pump in.	skeletal muscle rel.	sodium chloride	thyroid hormones	vitamins	threshold
AKUSSI joint pain	0.013	0.116	0.606	0.654	0.638	1.000	0.645	0.504	0.053	0.013
AKUSSI spinal pain	0.449	1.000	0.020	0.499	0.019	1.000	0.143	0.506	0.277	0.019
KOOS pain	1.000	1.000	**0.004**	0.232	**0.014**	0.330	0.613	0.294	**0.038**	0.054
KOOS symptoms	0.594	1.000	0.067	0.128	0.009	0.408	0.594	0.782	1.000	0.009
KOOS daily living	0.616	0.552	0.087	0.667	**0.006**	0.358	0.616	0.266	0.765	0.022
KOOS sport	0.727	0.262	**0.002**	0.057	**0.004**	1.000	0.516	0.851	0.821	0.005
KOOS QOL	0.708	0.239	**0.004**	0.306	**0.018**	0.549	1.000	0.250	0.422	0.026
HAQ-DI	0.163	0.522	**0.061**	0.091	**0.011**	0.617	0.623	0.336	0.696	0.011
hap-VAS	0.202	1.000	**0.003**	0.202	**0.000**	0.688	0.094	0.373	0.252	0.007

Supplementary Table S3. Result of the Fisher's exact test between every drug and QoL score; the last column represents the threshold computed with the Benjamini-Hochberg procedure; the drugs which show a significant correlation are highlighted with bold characters.

1. McDonald, J. *Handbook of Biological Statistics*; Sparky House Publishing, Baltimore, Maryland, 2014.
2. Fisher, R.A. On the Interpretation of chi squared from Contingency Tables, and the Calculation of P. *Journal of the Royal Statistical Society* **1922**, *85*, 87–94.
3. Benjamini, Y.; Hochberg, Y. Controlling the False Discovery Rate: A Practical and Powerful Approach to Multiple Testing. *Journal of the Royal Statistical Society. Series B (Methodological)* **1995**, *57*, 289–300.
